# The Anatomy and Surgery of Inguinal and Femoral Hernia

**Published:** 1834-04-01

**Authors:** 


*The Anatomy and Surgery of Inguinal and Femoral Hernia. Illustrated by Plates drawn from Nature, and interspersed with Practical Remarks.* By E. W. TUSON, F.L.S., Assistant Surgeon to the Middlesex Hospital, &c.—London, 1834. Folio, pp. 16, and three Plates.

WE do not know who was the inventor of dissected plates: the earliest specimen of these stratified engravings that has fallen into our hands was published at Amsterdam, in 1645. It is called Pinax Microcosmographicus, and the title-page sets forth that it had been written by Stephen Michael Spacher, of the Tyrol, for the benefit of physicians, surgeons, and apothecaries; and that it was then translated "into our mother-tongue," (the Dutch, to wit,) and artistically engraved *(artificiose sculptus)* by Cornelius Dancker. The plates are good, considering the period when they were executed, and are adorned with many quaint devices; the explanations are in Latin and Dutch.

But we must now speak of Mr. Tuson's book, whose claims to praise are of a far higher order. It is a very handsome work, and does great credit to the author, as well as to the artists whom he has employed. The upper part of the first plate contains the anatomy of inguinal hernia, and the lower part of the same plate the anatomy of femoral hernia. The second plate gives the dissection of an oblique inguinal hernia, and in the third plate we find the dissection of a femoral hernia.

This work will be of especial service to practitioners in the country, who may not always be able to procure subjects, when they wish to revive their faded recollections of surgical anatomy.

